# Factors affecting bone mineral density in multiple sclerosis patients

**Published:** 2013

**Authors:** Azin Ayatollahi, Mohammad Reza Mohajeri-Tehrani, Shahriar Nafissi

**Affiliations:** 1 Department of Neurology, School of Medicine, Tehran University of Medical Sciences, Tehran, Iran; 2 Endocrinology and Metabolism Research Center, Tehran University of Medical Sciences, Tehran, Iran

**Keywords:** Multiple Sclerosis, Bone Mineral Density, Osteoporosis, Quality of Life, Disability

## Abstract

**Background:**

Multiple sclerosis (MS) is a demyelinating disease which can cause many disabilities for the patient. Recent data suggests that MS patients have higher risk for osteoporosis. This study was performed to investigate if the osteoporosis prevalence is higher in MS patients and to determine the possible factors affecting bone mineral density (BMD).

**Methods:**

51 definite relapsing-remitting MS patients according to McDonald's criteria (45 females, 6 males aged between 20 and 50 years) participated in this study. The control group included 407 females aged from 20 to 49 years; they were healthy and had no history of the diseases affecting bone metabolism. Femoral and lumbar BMD were measured by Dual Energy X-ray Absorptiometry (DXA). The disability of MS patients was evaluated by Expanded Disability Status Scale (EDSS). The patient's quality of life was evaluated by the validated Persian version of multiple sclerosis impact scale (MSIS-29).

**Results:**

Patients’ mean age was 36 ± 3.3 years and their mean disease duration was 8.7 ± 1.7 years. The mean EDSS score and the mean body mass index (BMI) of the patients were 3 ± 0.9 and 23.5 ± 2.3 kg/m^2^, respectively. 29% of the patients had never been treated by ß-interferon and 6% of them had not received glucocorticoids (GCs) pulses since their MS had been diagnosed. 26% of the patients had a history of fracture.18% of our patients were osteoporotic and 43% of them were osteopenic. Femoral BMD was significantly lower among MS patients than age matched controls (P < 0.001), but lumbar BMD showed no difference. There was no correlation between administration of GCs pulses, interferon and BMD; however, we found a significant correlation between EDSS score, quality of life (QoL), disease duration and BMD of both site.

**Conclusion:**

As a result of this study, bone loss inevitably occurs in MS patients. The major factor of BMD loss is immobility. Osteoporosis should be managed as part of MS patients’ treatment protocols.

## Introduction

Multiple sclerosis, an inflammatory demyelinating condition, is one of the most common chronic diseases of central nervous system, which can have relapsing remitting or progressive course. Young adults are the most victims of this disease; mainly, the sufferers are women.^1^ By involvement of the sensory, motor, cerebellar and visual system, it will cause disability, which in turn may cause gait impairment and imbalance, and lead to falls and fracture.^2^ Recent data suggests that multiple sclerosis (MS) patients have higher risk for osteoporosis than age matched controls.^3–5^ Factors such as disability, quality of life (QoL), vitamin D deficiency, and administration of high dose Glucocorticoids (GCs) may be responsive for low bone density in MS patients.

The combination of a tendency for falls and negative factors that influence bone mineral density (BMD) makes MS patients a potential high-risk population for osteoporotic fractures, which maybe the cause of morbidity and even mortality for them. Despite the facts that there are many known factors affecting BMD and leading to osteoporotic fractures in MS patients, the literature background on this issue is limited. Understanding the reasons associated with a decreased bone mass in MS patients will help in defining the appropriate therapeutic intervention, which was the aim of the present study.

## Materials and Methods

### Participants

This study was carried out in the MS clinic of Shariati Hospital, one of Tehran University of Medical Sciences’ Educational Hospitals. 51 definite relapsing remitting MS patients, according to McDonald's criteria, aged between 20 to 50 years have participated in this study.^6^ Patients in the attack phase of MS and those with other disorders affecting bone mineral metabolism (e.g. hyperthyroidism, hyperparathyroidism, end stage renal disease (ESRD), malabsorption, Rheumatologic disease) were excluded.

The functional status and disability of MS patients was evaluated by the Expanded Disability Status Scale (EDSS).^7^ Furthermore, MS related disability was classified into three groups. The first group was patients with mild disability (EDSS: 0-3.5). The second and third group had moderate disability (EDSS: 4-5.5) and severe disability (EDSS: 6-8), respectively. The total, physical, and psychological quality of life of MS patients was evaluated by the validated Persian version of multiple sclerosis impact scale (MSIS-29).^8–17^ The score ranged from 0 to 100; besides, the higher score showed the lower QoL.

Patients’ body mass index (BMI) was calculated. The patients had not received oral prednisolone or other kinds of GCs within the last year, with the exception of GCs pulses.

The number of times that a patient received methylprednisolone pulse therapies was asked. The duration of the disease from the time when the first symptom occurred and the administration of ß Interferon were reported. Also, patients had no history of cigarette smoking or alcohol consumption. The group included 407 females aged between 20 to 49 years. These women were healthy; moreover, they did not have any history of menstrual problem, endocrine, renal, rheumatologic disease. Besides, they had never smoked cigarette or drank alcohol. These women were chosen randomly from different parts of Tehran. Their BMD were evaluated in order to assess the prevalence of osteoporosis among young healthy women in endocrinology research center of Shariati Hospital.

The patients’ BMD also were measured in Shariati Hospital BMD center by Dual Energy X-ray Absorptiometry (DXA). Both femoral and lumbar spine (L_1_–L_4_) BMD were measured. 2 biochemical markers of the bone, serum calcium and 25 (OH) vitamin D in the endocrinology lab of Shariati Hospital were measured. Serum Calcium was measured by automatic standard laboratory methods while 25 (OH) vitamin D was studied using an Institute National Radio Elements (INR) kit by a competitive protein binding radioimmunoassay (RIA) method. This study was approved by the Local Ethics Committee of the University.

### Statistical Analysis

In order to evaluate data, descriptive statistics(mean ± standard error, 95% confidence interval) were used. Furthermore, mean BMD values of the patients and the controls were compared by t-test.

For the MS patients, the correlation between BMD of femoral and lumbar site and the independent variables (EDSS, QoL, age, disease duration, BMI, history of fracture, serum calcium and 25 (OH) vitamin D) was analyzed by simple linear regression. T-test was used to compare mean BMD values between genders as well as BMD values among groups of receiver and non-receiver of interferon, Calcium-Vitamin D (Ca-D) supplements, and GCs pulses. In this study, one way variance analysis was used to compare mean BMD values among groups in terms of EDSS.

To compare BMD values among groups in terms of times which they receive GCs pulses, one way variance analysis (ANOVA) was used for femoral site with normal distribution. Since normality assumption was violated, Kruskal-Wallis test was used at the lumbar site. If there was a significant correlation, the multiple regression analysis by enter method was performed. It is worth mentioning that all of the analyses of this study were performed by the help of Minitab Software.

## Results

The estimated mean age of patients was 36 ± 3.3 years and mean disease duration was 8.7 ± 1.7 years. 88% of the patients were women. The mean EDSS score and the mean BMI of the patients was 3 ± 0.9 and 23.5 (with the range of 14.27 to 33.67), respectively. None of the female patients were menopause. 6% of the patients had not received GC pulses since their MS had been diagnosed. 29% of the patients had never been treated by interferon. 26% of the patients had a history of fracture.

The mean and 95% confidence interval of EDSS groups were as follows [mean ± SE]:

Mild disability: 41 (80%) patients, 2 ± 0.2, 95% CI 1.5-2.5

Moderate disability: 7 (14%) patients, 4.5 ± 0.2, 95% CI 4-5

Severe disability: 3 (6%) patients, 7 ± 0.5, 95% CI 6-8

The mean and 95% CI of the total aspects of QoL were 27.5 ± 2.4 (95% CI: 22.7-32.3), physical aspect of QoL mean was 26.4 ± 2.39 (CI: 21.8-31). Mean psychological aspect of QoL was 33.6 ± 2.5 (CI: 28.7-38.5).

All patients had normal range of serum calcium with the mean of 9.44 ± 0.1 (Normal range: 8.5-10.4). Mean 25 (OH) vitamin D was 30.75 ± 6.9. 12.5% of the patients had 25 (OH) vitamin D level below 10 mg/ml (vitamin D deficient).

In order to compare BMD of case and control group, 44 MS female patients were compared with the control group with the mean age of 36.5 ± 4 and the mean BMI of 23.6 ± 2.3. As the male MS patients in the study were very rare and included only 6 patients, they were omitted from the comparison. The overall rates of Osteoporosis and Osteopenia in MS patients were 18% and 43%, respectively. The result of the comparison was as follows: at femoral site the mean BMD of MS patients was significantly lower than the control (P < 0.001), but at the lumbar site, mean BMD did not show any significant difference (P = 1).

### Relationships between BMD and other clinical variables

Z-score was used for comparison. After the analysis, the following relations were seen: A significant association between femoral and lumbar BMD and disease duration (P < 0.001). BMD values were significantly lower in MS patients with higher EDSS score at both sites (P < 0.001). ANOVA showed no difference between femoral BMD of 3 groups of EDSS (P = 0.172) and also lumbar BMD (P = 0.097). The correlation between total and physical aspects of QoL and BMD of both sites was statistically significant (P < 0.001). Psychological aspects of QoL had significant correlation with femoral BMD (P < 0.001) but had no correlation with lumbar BMD (P = 0.166). Patients’ age and BMD of both sites had significant correlation (P < 0.001). There was a significant correlation between age and EDSS (P = 0.008, r = 0.36 Pearson correlation). EDSS and physical aspects of QoL were significantly correlated (P < 0.001, r = 0.68).

For controlling the effects of age and physical aspect of QoL on EDSS, multiple regression analysis was performed. As the EDSS was strongly related to QoL (physical) and age, neither of our variants explains significantly more variation than the other.

Femoral site: P (age) = 0.387, P (physic) = 0.06, P (EDSS) = 0.866

Lumbar site: P (age) = 0.061, P (physic) = 0.05, P (EDSS) = 0.621

After the regression analysis the following were seen: BMI had significant positive correlation with BMD of both sites. [P (femoral site) < 0.001, P (lumbar) = 0.027]. History of fracture had significant negative correlation with BMD of both sites. [P (femur) = 0.002, P (lumbar) = 0.005]. There was no difference at lumbar site (P = 0.068) and femoral site (P = 0.109) between the two genders. After the analysis was performed for assessing the effects of GCs pulses and Interferon on the BMD, the following results were observed:

Neither of GCs pulses and Interferon showed any correlation with BMD of both sites.

GC Pulses: P (femur) = 0.588, P (lumbar) = 0.351

Interferon: P (femur) = 0.121, P (lumbar) = 0.108

There was no correlation between the number of times of GCs pulse administration (0, 1, 2, 3 and more) and BMD [P (femur) = 0.533, P (lumbar) = 0.678]

Serum calcium had no correlation with BMD of both sites [P (femur) = 0.134, P (lumbar) = 0.078]. 25 (OH) vitamin D had no correlation with femoral BMD (P = 0.271) but had a borderline negative correlation with lumbar BMD (P = 0.044). There was no correlation between Ca-D consumption and BMD of both sites [P (femur) = 0.815, P (lumbar) = 0.252, 51% of our patients had a history of Ca-D consumption during last year.

Table 1 shows BMD values of patients and control group at femoral and lumbar sites.

**Table 1:** Mean bone mineral density of patients and control group at femoral and lumbar sites

	Femoral		Lumbar	

Control	Multiple sclerosis	Control	Multiple sclerosis	Age group
1.045 ± 0.001	1.065 ± 0.003	0.974 ± 0.013	0.860 ± 0.022	20-29
1.069 ± 0.001	1.149 ± 0.005	0.971 ± 0.012	0.980 ± 0.027	30-39
1.059 ± 0.002	0.997 ± 0.001	0.953 ± 0.015	0.871 ± 0.020	40-49
1.071 ± 0.001	1.081 ± 0.002	0.966 ± 0.014	0.920 ± 0.024	Total

## Discussion

The results indicated that BMD was significantly lower in relatively young MS patients compared to healthy control group at femoral site, but not at the lumbar site. Our judgment is consistent with the expectation that BMD may be reduced in people with MS at femoral site. The same results have attained in other studies,^3–6^ for the femoral site; however, other studies showed a decreased BMD also at the lumbar site.

Progressive gait difficulty resulting in immobilization is relatively common in MS.^10^ BMD value was higher in the patients with lower EDSS score. These results show that as functional capacity decreases, femoral and lumbar BMD reduces. Other studies reported the same result.^4, 5^ EDSS, different aspects of QoL, age, and disease duration were determined as the main variables affecting both femoral and lumbar bone density in the MS patients. EDSS, physical aspect of QoL, and age were related variables, which had the same significant effect on BMD of both sites.

One of the important factors which is necessary for bone to keep its strength is mobility and physical activity. As the EDSS score and age increased, the physical QoL decreased, this factor is diminished in MS patients. Patient becomes more non-ambulatory; moreover, the risk of falling will increase because of the unbalancing caused by MS. All of this will result in osteoporotic fracture. This finding may necessitate some specific exercise program and drug intervention to increase the BMD.

A significant correlation between disease duration and BMD of lumbar and femoral site was identified. Nevertheless, the results from another study showed that it had correlation with femoral BMD, not lumbar site.^4^ There was no correlation between GC pulses, interferon consumption, and BMD. The same result was found in other studies.^3–5, 11^

Serum calcium and 25 (OH) vitamin D had no effect on BMD values in the patients. Single evaluation of bone turnover markers is not such a reliable method in the diagnosis of osteoporosis.^12^ As we found in this study, psychological aspect of QoL had significant correlation with femoral BMD. As bone pain and osteoporotic fracture would make the psychological QoL worse, it is important to prevent osteoporosis.

In our study there was no difference between gender's BMD. Weinstock-Guttman et al.^5^ reported that osteoporosis has high prevalence in male MS patients. So, this group of patients need more attention.

Our correlation analysis suggested that higher BMI values had a beneficial effect on BMD of femoral and lumbar sites. Osteoporosis is one of the few conditions in which excess body weight is protective^14^ and the association between BMD and BMI may contain contributions from the increased mechanical loads on bone at higher BMI and from metabolic and hormonal changes(e.g. leptin) related to obesity.^15, 16^

In conclusion, bone loss inevitably occurs in MS patient. As a result, they are the high risk group for falling and fracture. Immobility, patient's disability and duration of disease are the major factors predisposing MS patients to osteoporosis. In addition, QoL is reduced in MS patients with osteoporosis; so, diagnosis and management of osteoporosis should be part of the MS patient treatment protocols.

As the number of the patients was limited, especially the male patients and the course of disease in the entire patients’ group was RRMS, the results of this study might be considered preliminary and further studies clarifying the importance of this topic should be undertaken.
